# The mTOR Pathway Controls Cell Proliferation by Regulating the FoxO3a Transcription Factor via SGK1 Kinase

**DOI:** 10.1371/journal.pone.0088891

**Published:** 2014-02-18

**Authors:** Shunsuke Mori, Shigeyuki Nada, Hironobu Kimura, Shoji Tajima, Yusuke Takahashi, Ayaka Kitamura, Chitose Oneyama, Masato Okada

**Affiliations:** 1 Department of Oncogene Research, Research Institute for Microbial Diseases, Osaka University, 3-1 Yamadaoka, Suita, Osaka, Japan; 2 Laboratory of Epigenetics, Institute for Protein Research, Osaka University, 3-2 Yamadaoka, Suita, Osaka, Japan; Yokohama City University School of Medicine, Japan

## Abstract

The mechanistic target of rapamycin (mTOR) functions as a component of two large complexes, mTORC1 and mTORC2, which play crucial roles in regulating cell growth and homeostasis. However, the molecular mechanisms by which mTOR controls cell proliferation remain elusive. Here we show that the FoxO3a transcription factor is coordinately regulated by mTORC1 and mTORC2, and plays a crucial role in controlling cell proliferation. To dissect mTOR signaling, mTORC1 was specifically inactivated by depleting p18, an essential anchor of mTORC1 on lysosomes. mTORC1 inactivation caused a marked retardation of cell proliferation, which was associated with upregulation of cyclin-dependent kinase inhibitors (CDKIs). Although Akt was activated by mTORC1 inactivation, FoxO3a was upregulated via an epigenetic mechanism and hypophosphorylated at Ser314, which resulted in its nuclear accumulation. Consistently, mTORC1 inactivation induced downregulation of serum- and glucocorticoid-inducible kinase 1 (SGK1), the kinase responsible for Ser314 phosphorylation. Expression of FoxO3a mutated at Ser314 suppressed cell proliferation by inducing CDKI expression. SGK1 overexpression suppressed CDKI expression in p18-deficient cells, whereas SGK1 knockdown induced CDKI expression in wild-type cells, resulting in the suppression of cell proliferation. These results suggest that mTORC1, in coordination with mTORC2, controls cell proliferation by regulating FoxO3a gene expression and SGK1-mediated phosphorylation of FoxO3a at Ser314.

## Introduction

The mechanistic (or ‘mammalian’) target of rapamycin (mTOR) is a Ser/Thr kinase that regulates key cellular functions related to the promotion of cell growth and metabolism [1]. mTOR kinase functions as a component of two large complexes, mTORC1 and mTORC2, each of which contains specific regulatory proteins: mTORC1 contains Raptor [2] and PRAS40 [3], whereas mTORC2 contains Rictor [4], mSin1 [5], and Protor [6]. mTORC1 is preferentially inhibited by the macrolide rapamycin via an interaction with FKBP12, although the mechanism remains unclear [7], [8]. The functions and regulation of mTORC1 have been better characterized than those of mTORC2 [9].

mTORC1 activity is regulated by growth factors and nutrients. Activation of receptor tyrosine kinases by growth factors, such as insulin and insulin-like growth factor, stimulates Akt kinase via activation of phosphoinositide 3-kinase (PI3K). Activated Akt phosphorylates and inactivates TSC1/2, a GTPase-activating protein (GAP) for Rheb GTPases, resulting in activation of mTORC1 [10]. Activated mTORC1 phosphorylates eukaryotic translation initiation factor 4E binding protein 1 (4E-BP1) and S6 kinase 1 (S6K1), thereby promoting protein synthesis [11], [12]. mTORC1 also promotes lipid biogenesis and metabolism, and suppresses autophagy by regulating several other downstream effectors, such as TFEB, SREBP-1, HIF1α, and ULK-Atg13 [9], [13]. The activation of mTORC1 also leads to the inactivation of growth-factor signaling by closing the negative-feedback loop mediated by S6K1 [14], mTORC1 [15], and growth factor receptor-bound protein 10 (Grb10) [16], [17].

The activation of mTORC1 by nutrients is achieved on the surface of lysosomes [18], [19]. Amino acids supplied to starved cells are sensed by vacuolar ATPase (v-ATPase) on lysosomes, resulting in activation of Rag GTPase via Ragulator, a lysosomal scaffold protein complex with guanine nucleotide exchange factor (GEF) activity [19]. Activated Rag GTPase recruits and activates mTORC1 at the lysosomal surface via Rheb [20]. The Ragulator complex consists of five small proteins: p18, p14, MP1, HBXIP, and C7orf59. One of these, p18, has a fatty-acyl modification and serves as an essential anchor of the complex to the lysosomal membrane. We previously identified p18 as a membrane anchor of the p14/MP1 complex on late endosomes/lysosomes [21], and subsequently showed that p18 plays a crucial role in regulating mTORC1 function in lysosome biogenesis and maturation processes [22], [23].

Although the functions and regulatory mechanisms of mTORC2 remain unclear, interplay between the mTORC1 and mTORC2 pathways is crucial for control of cell proliferation and homeostasis. When cells are stimulated by growth factors, mTORC2 phosphorylates Akt at a specific site to facilitate its full activation by PDK1 [24]. Activated Akt contributes to activation of mTORC1 via TSC1/2, and also directly promotes cell growth by suppressing gene expression of cyclin-dependent kinase inhibitors (CDKIs), e.g., p27^Kip1^ and p21^Cip1^, and pro-apoptotic molecules, e.g., Bcl2-family proteins and Fas ligand [25]. This Akt-dependent survival function is mediated by the Fork head box O (FoxO) family of transcription factors, which consists of FoxO1, 3, 4, and 6 [26]. FoxO proteins function as key downstream effectors of growth-factor receptors, and are involved in the regulation of diverse cellular processes, including cell proliferation, apoptosis, longevity, cancer, and the cell cycle. Akt suppresses FoxO protein function by phosphorylating the transcription factor at critical sites required for export from the nucleus and degradation [26]. These observations indicate that mTOR signaling regulates cell growth and homeostasis by coordinating the interplay between mTORC1, mTORC2, Akt, and FoxO proteins, although the underlying molecular mechanisms remain to be clarified [27].

Previously, we showed that ablation of p18 induced dramatic growth retardation even under nutrient-rich conditions [21], indicating that mTORC1 plays crucial roles in controlling cell proliferation. However, the signaling pathways leading to growth arrest remain unknown. To address this issue, we analyzed the molecular circuits controlled by mTORC1 using p18-deficient cells. Because chronic treatment with rapamycin disrupts mTORC2 action in some cell types [8], p18-deficient cells are useful for dissecting the function of mTORC1 specifically, because the functions of other components of mTOR complexes are not affected in these cells. In this study, we found that inactivation of mTORC1 promoted the nuclear function of FoxO3a by activating its gene expression through an epigenetic mechanism, as well as by suppressing phosphorylation of FoxO3a at Ser314, a site required for nuclear export mediated by serum- and glucocorticoid- inducible kinase 1 (SGK1). Our findings suggest that mTORC1 coordinates with mTORC2 to control cell proliferation by regulating the nuclear function of FoxO3a.

## Materials and Methods

### Reagents and antibodies

The following reagents and antibodies were obtained commercially: MG-132 (Calbiochem), anti-Akt1, anti-phospho-Akt1 (Thr308, Ser473), anti-p44/42 (Erk1/2), anti-phospho-p44/42 (Erk1/2) (Thr202/Tyr204), anti-MEK1/2, anti-phospho-MEK1/2 (Ser 217/221), anti-mTOR, anti-phospho-mTOR (Ser2448), anti-p70S6K, anti-phospho-p70S6K (Thr389), anti-Rictor, anti-Raptor, anti-PRAS40, anti-phospho-PRAS40 (Thr246), anti-4E-BP1, anti-phospho-4E-BP1 (Thr 70), anti-FoxO1, anti-phospho-FoxO1 (Thr24 and Ser256), anti-FoxO3a, anti-phospho-FoxO3a (Ser253 and Thr32), anti-TSC2, anti-phospho-TSC2 (Thr1462), anti-SGK1, anti-SGK3 (Cell Signaling Technology), anti-p21^Cip1^ (Santa Cruz Biotechnology); anti-p27^Kip1^ (BD Biosciences), anti-cyclin D1 (MBL), and anti-β-tubulin (Sigma). Anti-phospho-FoxO3a Ser314 antibodies were a generous gift from Dr. Michael E. Greenberg (Harvard Medical School, Boston).

### Cell culture

*p18^fl/fl^* mouse embryonic fibroblasts (MEFs) were established from a p18-floxed mouse [22]. *p18^-/-^* cell lines were established by transfecting *p18^fl/fl^* MEFs with an integrase-defective Cre vector, a gift from Dr. Masahito Ikawa. Cells were cultured in Dulbecco's modified Eagle's medium (DMEM) supplemented with 10% fetal bovine serum, and grown at 37°C in a humidified atmosphere containing 5% CO_2_.

### Growth assay and flow cytometry analysis

For cell-growth assays, cells were seeded in 96-well plates (500 cells/well). Cell growth was measured using the WST-1 Cell Proliferation Reagent (Roche) at the indicated time points. For FACS analysis, cells were plated onto 60-mm dishes at a density of 8×10^5^ cells per dish. After 24 h, cells were harvested and stained with propidium iodide using the Cycle TEST™ PLUS DNA Reagent Kit, and acquisition was carried out on a FACScan flow cytometer (Becton Dickinson).

### Expression constructs

All gene-transfer experiments were carried out using the pCX4 series of retroviral vectors [28]. cDNA constructs were generated by the polymerase chain reaction (PCR) using mouse cDNA as a template. The p18ΔN5-CAAX mutant was generated by deleting the five N-terminal amino acids of p18 and adding the K-Ras CAAX motif (KHKEKMSKDGKKKKKKSKTKCVIM) to the C-terminus. FoxO3a and the short and long forms of SGK1 were cloned into a vector containing a hemagglutinin (HA) tag on the 5′ side of the cloning site. FoxO3a point mutants were generated by PCR-based mutagenesis. Mouse SGK1 lentiviral shRNA duplexes and non-target shRNA control (SHC202) were purchased from Sigma. The Mouse SGK1-shRNA sequences used was CGGCTGAGATGTACGACAATA.

### Real-time PCR analysis

Total RNA was extracted using Sepasol®-RNA Super G (Nacalai Tesque), and then reverse transcribed by extension with random hexamer primers, using the Transcriptor First Strand cDNA Synthesis Kit (Roche). qRT-PCR was performed using THUNDERBIRD® SYBR® qPCR Mix (TOYOBO) with the following primers: β-tubulin (Forward; TATGTACCTCGGGCCATCC, Reverse; TTATTTCCTGCACCACTCTGG), FoxO3a (Forward; GATAAGGGCGACAGCAACAG, Reverse; CATTCTGAACGCGCATGA), FoxO1 (Forward; CTTCAAGGATAAGGGCGACA, Reverse; GACAGATTGTGGCGAATTGA), Rictor (Forward; CACAAGCCCTTCGCTTAGTC, Reverse; GTTCACAGATGATGGCGATG), p27^Kip1^ (Forward; GTTAGCGGAGGCAGTGTCCA, Reverse; TCTGTTCTGTTGGCCCTTTT), and SGK1 (Forward; GGACTACATTAATGGTGGAGAGC, Reverse; AGAATCGAGCCCGTGGTT).

### Immunofluorescence

Cells were plated onto fibronectin-coated glass coverslips in 24-well culture plates. The cultures were rinsed once with cold phosphate-buffered saline (PBS), and then fixed for 30 min with 4% paraformaldehyde in PBS. After three rinses with IF Wash buffer (0.005% saponin in PBS), cells were permeabilized for 30 min with 0.25% saponin in PBS, and then blocked for 30 min with Blocking buffer (0.1% saponin, 1% BSA in PBS). The blocked coverslips were incubated overnight at 4°C with primary antibodies in Blocking buffer, rinsed three times with Wash buffer, and incubated for 3 h with secondary antibodies in Blocking buffer. The washed coverslips were mounted on glass slides using Prolong Gold (Invitrogen). Fluorescence was observed using an Olympus IX81 confocal microscope controlled by FluoView FV1000 software. The intensity of the signal for FoxO3a was determined by using MetaMorph software (Universal Imaging).

### Western blotting

Cells were washed with PBS and lysed in cell lysis buffer (0.1% SDS, 0.1% Sodium deoxycholate, 25 mM Tris-HCl [pH 7.4], 150 mM NaCl, 1 mM EDTA, 1% NP-40, 50 mM NaF, 1 mM Na_3_VO_4_, and protease inhibitor cocktail [Nacalai Tesque]) on ice. Equal amounts of total protein were separated by SDS-PAGE and transferred onto polyvinylidene difluoride (PVDF) membranes. Membranes were blocked and incubated with primary antibodies, followed by incubation with HRP-conjugated secondary antibodies. Signals from immunopositive bands were visualized on X-ray film using Immuno Star Zeta (Wako, Tokyo, Japan). Representative blots obtained from at least three independent experiments are shown. Subcellular fractionation was performed according to a previously described method [29]. Cells were plated on 100-mm dishes at a density of 5×10^5^ cells per dish. After 2 days, the cells were harvested and separated into cytoplasm and nuclear fractions.

### Bisulfite sequencing

Sodium bisulfite modification of genomic DNA was conducted using the EpiTect® Bisulfite Kit (QIAGEN). Bisulfite-treated DNA was used as the template for PCR using the following primers: 5′-GGTTTTGGGTAATTAAGGAAATGTT-3′ and 5′-ACTCCTCTACCAACCCTCTCTAAAC-3′. Amplified products were subcloned using the TOPO-TA cloning system (Invitrogen). Plasmid DNAs from ten insert-positive clones were isolated and sequenced.

### Reporter constructs and luciferase assay

The mouse *Foxo3* intronic region (Full, +1928/+2914; Del-1, +2145/+2914; Del-2, +2245/+2914; Del-3, +2381/+2914) was amplified by PCR from the RPCI-23 C57BL/6J Mouse BAC Library (BAC/PAC Resources), and then subcloned into the luciferase reporter plasmid pGN-P2 (Toyo Ink, Tokyo, Japan). For luciferase assays, cells were co-transfected with an intronic-region construct along with the control plasmid pRL-TK (Toyo Ink). Cells were harvested 24 h after transfection, and luciferase activities were measured using the PicaGene Dual Sea Pansy Luminescence Kit (Toyo Ink).

## Results

### Inactivation of mTORC1 by the loss of p18 causes growth arrest

**Figure 1:**
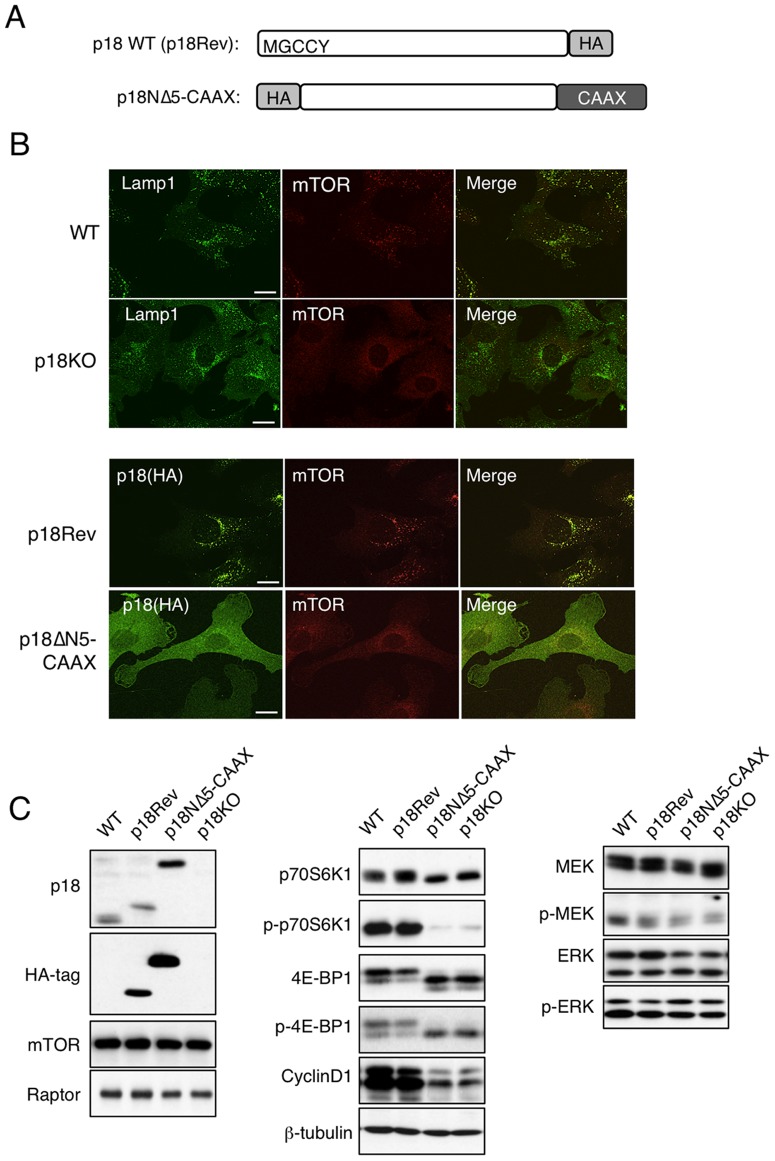
Lysosomal localization is required for mTORC1 activation. (**A**) Schematic structures of HA-tagged wild-type p18 (p18Rev) and a p18 mutant targeted to the plasma membrane (p18NΔ5-CAAX). (**B**) Immunofluorescence analyses of localizations of p18 and mTOR. WT and p18KO cells were stained for Lamp1 (green), a lysosome marker, and mTOR (red) (upper panels). p18Rev and p18NΔ5-CAAX cells were stained for p18 (HA) and mTOR (lower panels). Scale bars: 10 µm. (**C**) Western-blot analyses to detect the indicated signaling molecules, using total cell lysates from WT, p18Rev, p18NΔ5-CAAX, and p18KO cells.

To dissect the role of lysosomal mTORC1 in cell-cycle progression, we generated p18-knockout cells (p18KO) from *p18^flox/flox^* MEFs [22] using an *in vitro* Cre-loxP system. We also prepared a revertant cell line that re-expresses p18 in p18KO cells (p18Rev), for use as a control. Furthermore, to verify the significance of lysosomal localization of mTORC1, we introduced a mutant p18 that lacks the five N-terminal amino acids required for lysosomal localization, but contains the K-Ras CAAX motif at the C-terminus (p18NΔ5-CAAX), into p18KO cells (Figure 1A). In WT cells, mTOR was localized to perinuclear Lamp1-positive lysosomes, whereas in p18KO cells it was diffusely distributed in the cytoplasm (Figure 1B). Re-expressed p18 was widely colocalized with mTOR on lysosomes. By contrast, p18NΔ5-CAAX was distributed in the cytoplasm and on the plasma membrane, resulting in delocalization of mTOR from lysosomes (Figure 1B). These observations confirm that p18 is required for lysosomal localization of mTOR.

The activity of mTORC1 was assessed by determining phosphorylation of two mTORC1 substrates, p70S6K1 and 4E-BP1 (Figure 1C). mTORC1 activity was strongly inhibited by delocalization of mTOR from lysosomes, although the expression of mTOR and Raptor was unaffected. As a consequence, protein expression of growth-promoting cyclin D1 was dramatically suppressed (Figure 1C). By contrast, the loss of lysosomal p18 did not significantly affect the activity of the MAP kinase pathway (Figure 1, right panel), suggesting that Ragulator does not play a prominent role as a scaffold for MEK1 [30], [31], at least in these cells. These results demonstrate that lysosomal p18 is primarily involved in mTORC1 activation on lysosomes.

**Figure 2:**
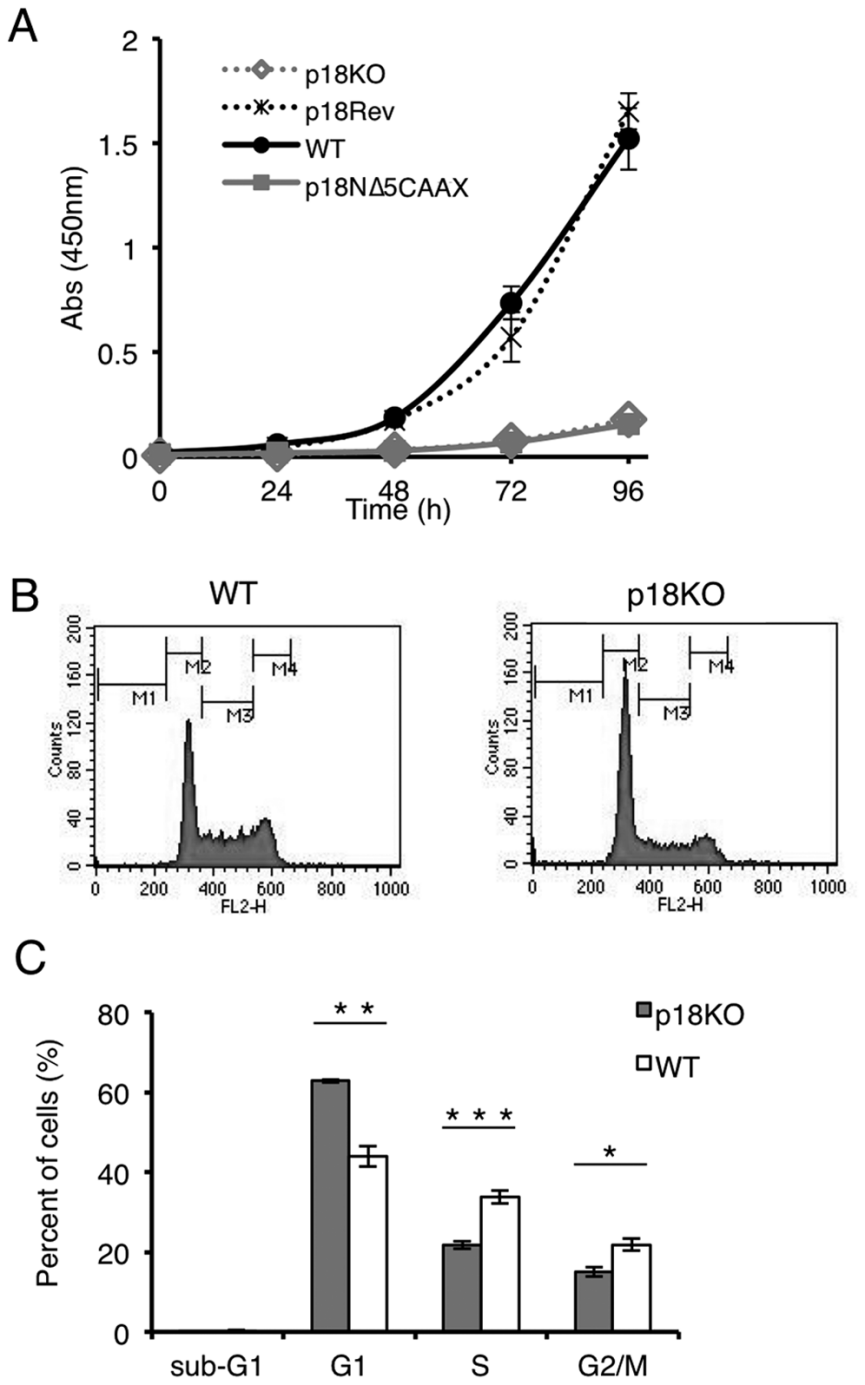
Inactivation of lysosomal mTORC1 causes growth arrest. (**A**) Cell proliferation of WT, p18Rev, p18NΔ5-CAAX, and p18KO cells analyzed by the WST-1 growth assay over the indicated time course. (**B**) WT and p18KO cells were analyzed for DNA content by flow cytometry. M1-4 indicates the following areas: M1; sub-G1, M2; G1, M3; S, M4; G2/M. (**C**) Quantitative data from FACS analysis. Means±SD were obtained from three independent assays. ***P<0.001, **P<0.01, and *P<0.05 (Student's *t*-test).

We then examined the effects of mTORC1 inactivation on cell proliferation, using the WST-1 assay (Figure 2A). p18KO and p18NΔ5-CAAX cells exhibited a dramatic reduction in cell proliferation rates relative to WT and p18Rev cells. Flow cytometry analysis of p18KO and WT cells revealed that a significant proportion of p18KO cells were arrested in G1-phase (Figures 2B and 2C). These findings demonstrate that induction of cell-cycle entry by p18-mediated activation of lysosomal mTORC1 is required for promotion of cell proliferation.

### Inactivation of mTORC1 upregulates mTORC2 and FoxO3a

**Figure 3:**
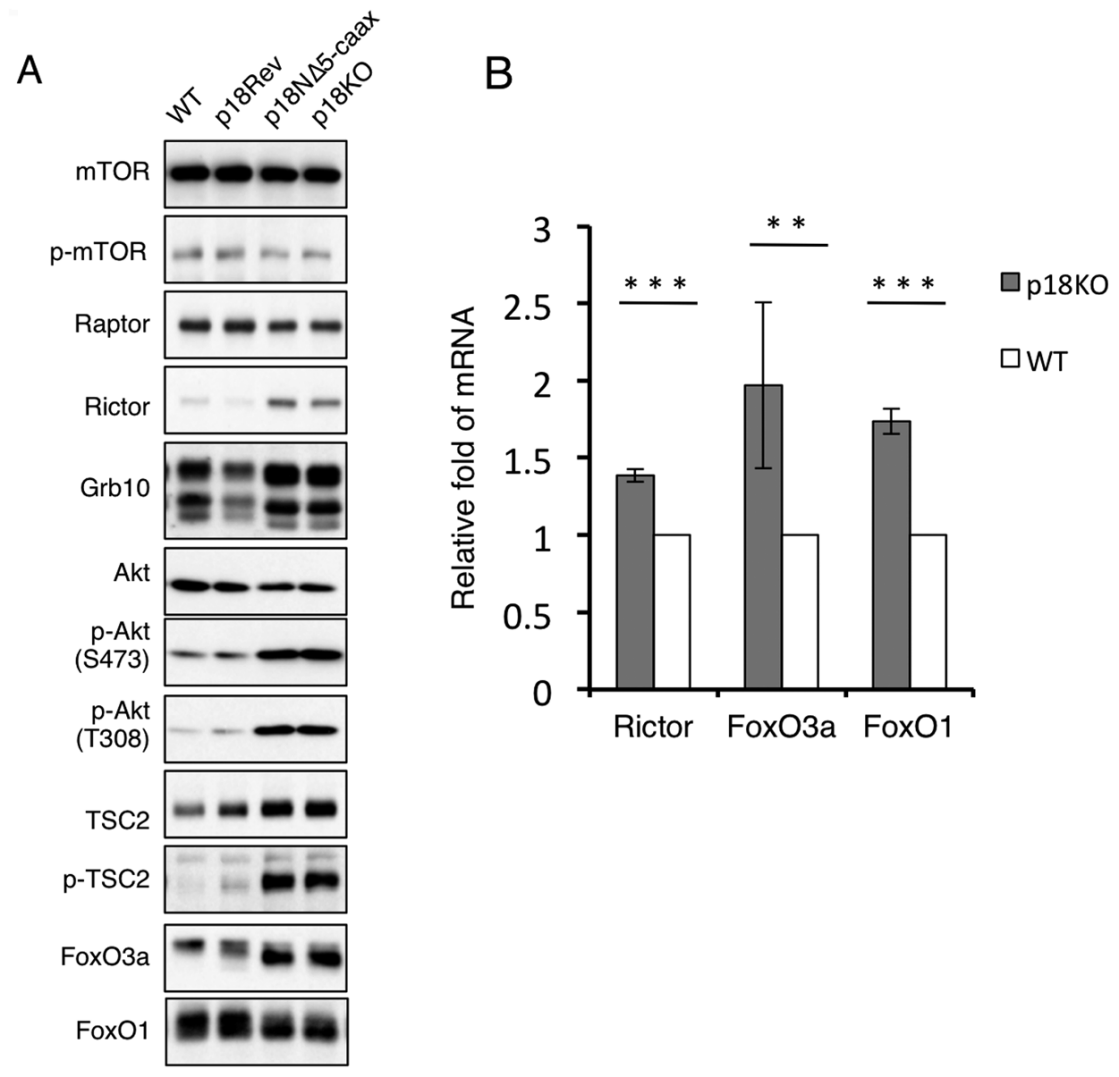
Inactivation of mTORC1 upregulates mTORC2 and FoxO3a. (**A**) Western-blot analyses to detect the indicated signaling molecules, using total cell lysates from WT, p18Rev, p18NΔ5-CAAX, and p18KO cells. (**B**) Expression levels of mRNAs encoding Rictor, FoxO3a and FoxO1 in p18KO and WT cells were determined by quantitative real-time PCR. Means±SD were obtained from three independent assays. ***P<0.001 and **P<0.01 (Student's *t*-test).

To address the molecular mechanisms by which lysosomal mTORC1 regulates cell proliferation, we investigated changes in signaling molecules upstream and downstream of the mTOR pathway (Figure 3A). Western-blot analyses of total cell lysates revealed that expression levels of mTOR and Raptor were not significantly influenced by inactivation of mTORC1, whereas protein and mRNA levels of Rictor were significantly upregulated (Figures 3A and B). Consistent with this, phosphorylation of Akt at Ser473, a site specifically phosphorylated by mTORC2 [24], was elevated, indicating that mTORC2 is activated by mTORC1 inactivation. Activated S6K1 phosphorylates Grb10, which mediates negative-feedback regulation of growth-factor signaling [17]. Indeed, inactivation of S6K1 by mTORC1 inactivation induced a mobility shift of Grb10, indicating dephosphorylation of this protein. Consequently, phosphorylation of Akt at Thr308, a PDK1 phosphorylation site [32], was elevated, probably due to activation of PI3K. Although the reason remains unknown, the Grb10 expression level was slightly decreased in p18Rev cells compared with WT cells. However, the phosphorylation level of Akt Thr308 in these cells was almost comparable with that in WT cells, suggesting that perturbation of Grb10 expression had no significant effect on the Akt pathway. Activation of Akt was further confirmed by the elevated phosphorylation of an Akt substrate, TSC2. These results suggest that inactivation of mTORC1 strongly activated Akt via activation of mTORC2 and abrogation of the negative-feedback inhibition mediated by Grb10. Activated Akt phosphorylates FoxO transcription factors and suppresses their nuclear function [26]. In p18KO cells, the levels of FoxO3a protein and mRNA were significantly upregulated (Figures 3A and B). Although the level of FoxO1 protein, a close relative of FoxO3a, appeared unchanged (Figure 3A), the FoxO1 mRNA level was significantly upregulated (Figure 3B). Furthermore, both FoxO3a and FoxO1 proteins exhibited apparent gel-mobility shifts (Figure 3A), as discussed further below. These findings suggest that the expression and modification of FoxO transcription factors are regulated via the p18-mTORC1 pathway. Based on the observations that FoxO3a protein is more markedly upregulated in p18KO cells than FoxO1 and that FoxO3a is widely expressed in various tissues [33], we hereafter focused on the regulatory mechanism for FoxO3a function.

**Figure 4:**
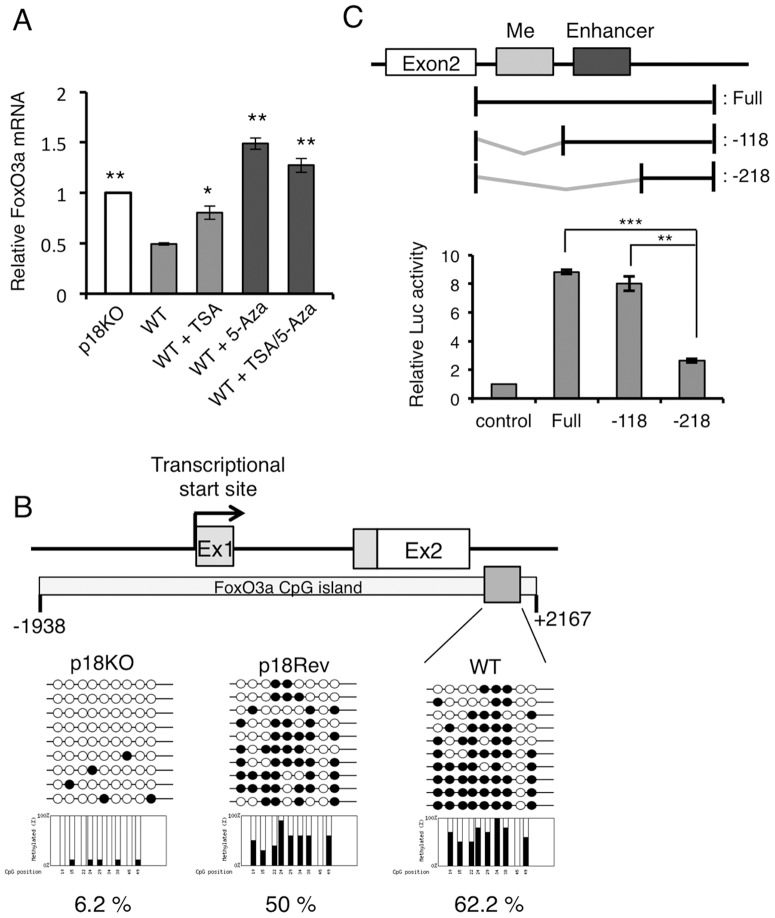
Expression of FoxO3a is regulated by DNA methylation. (**A**) WT cells were treated with trichostatin A (TSA), 5-aza-deoxycytidine (5-Aza), or a combination of TSA and 5-Aza for 48 h, and FoxO3a mRNA levels were determined by quantitative real-time PCR. Fold change in mRNA levels was calculated after normalization against β-tubulin mRNA (internal control). **P<0.01 and *P<0.05 (Student's *t*-test). (**B**) DNA methylation status of *FoxO3* CpG islands in p18KO, p18Rev, and WT cells was analyzed by bisulfite sequencing. The methylation status in the gray boxed region was determined for ten clones obtained from each cell line. Individual clones are indicated by lines with circles. Methylated and non-methylated cytosines are indicated by closed and open circles, respectively. (**C**) A series of fragments from the intronic region of the mouse FoxO3a gene were subcloned upstream of a luciferase reporter gene. Each construct was transfected into MEFs, and luciferase activity was measured and normalized against pRLTK activity. Normalized luciferase activity is expressed as means±SD (*n* = 3). ***P<0.001 and **P<0.01 (Student's *t*-test).

Because the upregulation of FoxO3a was a chronic event, we investigated the role of the epigenetic mechanisms for FoxO3a upregulation in p18KO cells. Treatment of WT cells with 5-aza-deoxycytidine (5-aza-dCA), an inhibitor of DNA methyltransferase, significantly upregulated the transcription of FoxO3a; trichostatin A (TSA), an inhibitor of histone deacetylase, also upregulated *Foxo3* transcription, albeit to a lesser extent (Figure 4A). This observation suggests that the expression of the *Foxo3* gene is affected by its DNA methylation status. Bisulfite sequencing analyses of *Foxo3* CpG islands revealed that DNA demethylation of a specific region at the 5′ end of the second intron occurred specifically in p18KO cells. Luciferase reporter assays revealed that the enhancer activity was located close to the 3′ end of the methylated region (Figure 4C). Thus, it is possible that DNA methylation in the adjacent region would interfere with enhancer activity, and hence inactivate the expression of *Foxo3* gene. These observations suggest that mTORC1 is involved in the regulation of gene expression by controlling the DNA methylation status of the *Foxo3* gene.

### Inactivation of mTORC1 induces nuclear accumulation of FoxO3a

**Figure 5:**
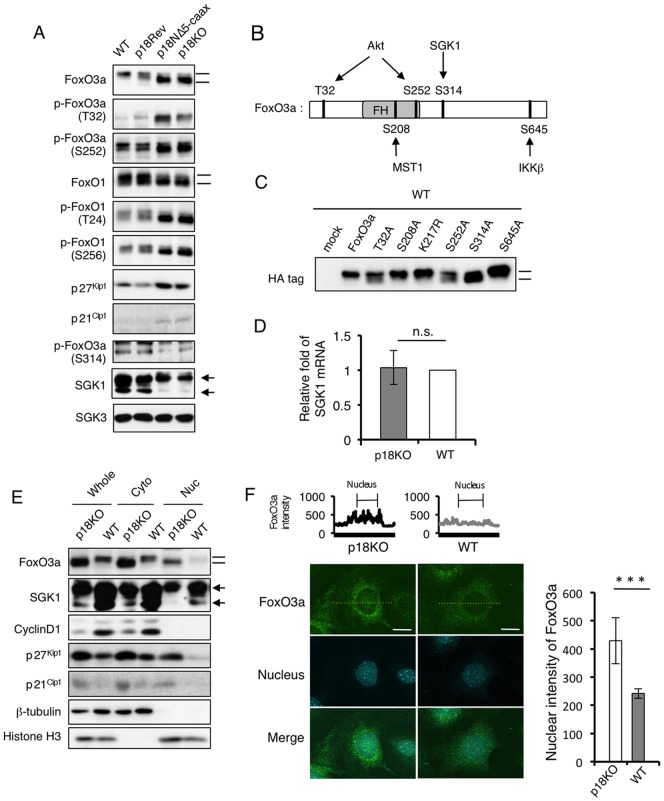
Inactivation of mTORC1 induces nuclear accumulation of FoxO3a. (**A**) Western-blot analyses to detect the indicated signaling molecules, using total cell lysates from WT, p18Rev, p18NΔ5-CAAX, and p18KO cells. The panels for FoxO3a and FoxO1 are the same panels used in Figure 3A. Mobility shifts of FoxO proteins are shown by bars. Locations of bands corresponding to long and short forms of SGK1 are indicated by arrows. (**B**) Schematic structure of FoxO3a. Sites of phosphorylation by the indicated kinases are shown. FH: Fork head domain. (**C**) HA-tagged FoxO3a constructs with point mutations at the indicated amino-acid positions were transiently expressed in WT cells, and their mobility shifts (indicated by bars) were analyzed by Western blotting. (**D**) Expression levels of mRNA encoding SGK1 in p18KO and WT cells were determined by quantitative real-time PCR. Means±SD were obtained from three independent assays. n.s.; not significant (Student's *t*-test). (**E**) Whole-cell lysates from p18KO and WT cells were separated into cytoplasmic and nuclear fractions, and the indicated proteins were detected by Western-blot analyses. β-tubulin and histone H3 represent control proteins for the cytoplasmic and nuclear fractions, respectively. (**F**) Immunofluorescence analysis for FoxO3a in p18KO and WT cells. Nuclei were visualized with propidium iodide (PI). Merged images are also shown. Scale bars: 10 µm. Upper graphs show the intensity of signals for FoxO3a obtained by scanning along the yellow dot lines. Right graph shows the statistic data of nuclear intensity of FoxO3a signals in p18KO and WT cells. Means±SD were obtained from 15 cells. ***P<0.001 (Student's *t*-test).

As mentioned above, we noticed that mTORC1 inactivation caused mobility shifts and hyperphosphorylation of Akt phosphorylation sites in FoxO3a and FoxO1 proteins (Figures 5A and 5B). Akt phosphorylates and inactivates FoxO proteins by causing them to translocate from the nucleus to the cytoplasm, which results in the promotion of cell proliferation via the suppression of the expression of CDKIs and pro-apoptotic molecules [25]. Surprisingly, however, expression of two CDKIs, p27^Kip1^ and p21^Cip1^, was increased by Akt-mediated FoxO phosphorylation (Figure 5A).

To resolve this apparent discrepancy, we investigated the phosphorylation status at Ser314, a site that is preferentially phosphorylated by SGK1, an Akt-related kinase that phosphorylates FoxO3a [34] (Figure 5B). Western-blot analysis using a site-specific antibody revealed that phosphorylation at Ser314 was decreased by mTORC1 inactivation, despite upregulation of FoxO3a protein expression (Figure 5A). To further confirm the reduced phosphorylation at Ser314, we overexpressed several point mutants of FoxO3a in WT cells and analyzed their mobility shifts (Figure 5C). Mutants lacking Akt phosphorylation sites (T32A and S252A) exhibited only moderate mobility shifts, whereas a mutant lacking the SGK1 site (S314A) exhibited a more dramatic mobility shift. Mutation at sites phosphorylated by MST1 or IKKβ did not affect mobility. These results demonstrate that the mobility shift of FoxO3a observed in mTORC1-inactivated cells was due to reduced phosphorylation at Ser314. Consistent with this, we found that protein expression of SGK1, but not SGK3, was markedly suppressed by mTORC1 inactivation, although SGK1 mRNA levels were unaffected (Figure 5A and 5D). As described below, SGK1 is expressed as several isoforms with distinct N-termini, generated by translation initiation at alternative sites [35]. The expression of the shortest form was clearly downregulated. These findings suggest that phosphorylation at Ser314, potentially mediated by SGK1, is crucial for the regulation of FoxO3a function.

To elucidate the contribution of mTORC1-mediated modification to the function of FoxO3a, we examined nuclear localization of FoxO3a in p18KO and WT cells. Subcellular fractionation analysis revealed that p18KO cells accumulated more nuclear FoxO3a protein, which lacks Ser314 phosphorylation, than WT cells (Figure 5E). Quantitative immunofluorescence analysis also revealed that FoxO3a was significantly accumulated in the nucleus of p18KO cells (Figure 5F). Accumulation of CDKIs in the nucleus was detected in p18KO cells. SGK1 was more abundantly localized to the nucleus in WT cells than in p18KO cells, indicating that it facilitates nuclear export of FoxO3a by phosphorylating Ser314. These observations suggest that hypophosphorylation at Ser314 is involved in nuclear accumulation of FoxO3a in p18KO cells.

### Hypophosphorylation of Ser314 promotes nuclear entry of FoxO3a

**Figure 6:**
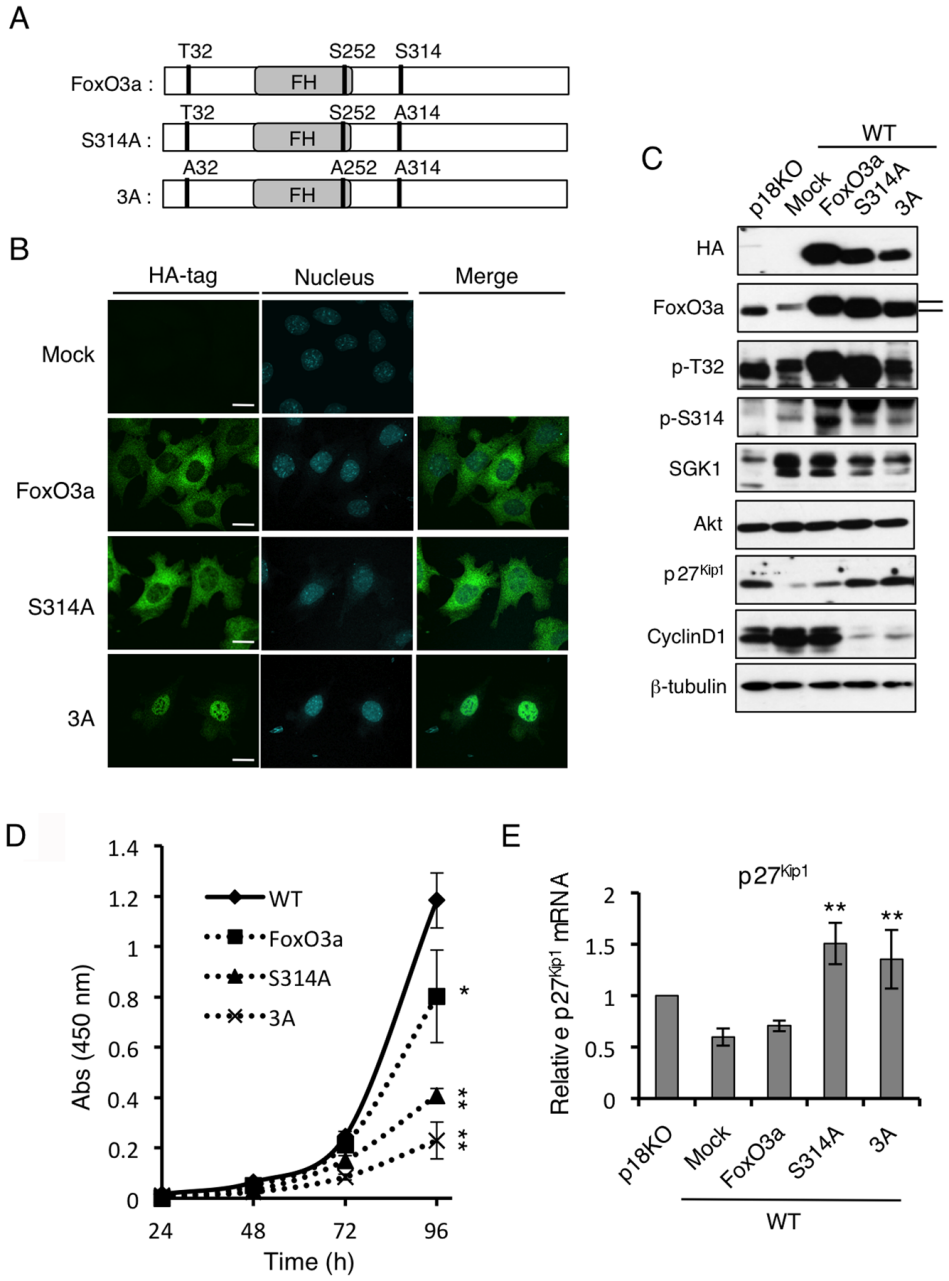
Nuclear localization of FoxO3a suppresses cell proliferation. (**A**) Schematic structures of WT FoxO3a, FoxO3a S314A mutant (S314A), and FoxO3a T32/S252/S314A triple mutant (3A). (**B**) Immunofluorescence analyses to determine localizations of HA-tagged WT FoxO3a, S314A, and 3A cells to determine FoxO3a (HA) localization. Merged images with PI staining are shown. Scale bars: 10 µm. (**C**) Western-blot analyses for the indicated molecules in p18KO cells, mock-treated WT cells, and WT cells expressing FoxO3a, S314A, or 3A. (**D**) Cell proliferation of WT cells and WT cells expressing WT FoxO3a, S314A, or 3A was analyzed by the WST-1 growth assay over the indicated time course. Means±SD were obtained from three independent assays. **P<0.01 and *P<0.05 (Student's *t*-test). (**E**) Expression level of p27^Kip1^ mRNA in cells used in (C) was determined by quantitative real-time PCR. Means±SD were obtained from three independent assays. **P<0.01, (Student's *t*-test).

To examine the role of Ser314 phosphorylation in the regulation of cell proliferation, we generated WT cell lines that stably overexpressed wild-type FoxO3a, the S314A mutant (S314A), or a mutant with triple alanine replacements at T32, S252, and S314 (3A) (Figure 6A). Immunofluorescence analysis revealed that whereas WT FoxO3a was mostly distributed in the cytoplasm, the S314A mutant was localized to both the cytoplasm and the nucleus (Figure 6B). This distribution pattern of the S314A mutant was consistent with that of FoxO3a in p18KO cells (Figure 5F), indicating that hypophosphorylation of Ser314 allows nuclear entry of FoxO3a even though other sites are hyperphosphorylated (Figure 6C). The 3A mutant was predominantly localized to the nucleus (Figure 6B), which supports the results of a previous observation showing that Akt-mediated phosphorylation contributes to the nuclear export of FoxO3a [33]. In both cases, the nuclear localization of FoxO3a mutants strongly suppressed cell proliferation (Figure 6D). Western-blot and real-time PCR analyses confirmed that expression of the S314A and 3A mutants resulted in elevated protein and mRNA levels of p27^Kip1^, and these levels were inversely correlated with cyclin D1 expression (Figures 6C and 6E). These findings suggest that the phosphorylation status of Ser314 is crucial for the regulation of the nuclear function of FoxO3a.

### SGK1 is involved in the regulation of cell proliferation via the FoxO3a-CDKI axis

**Figure 7:**
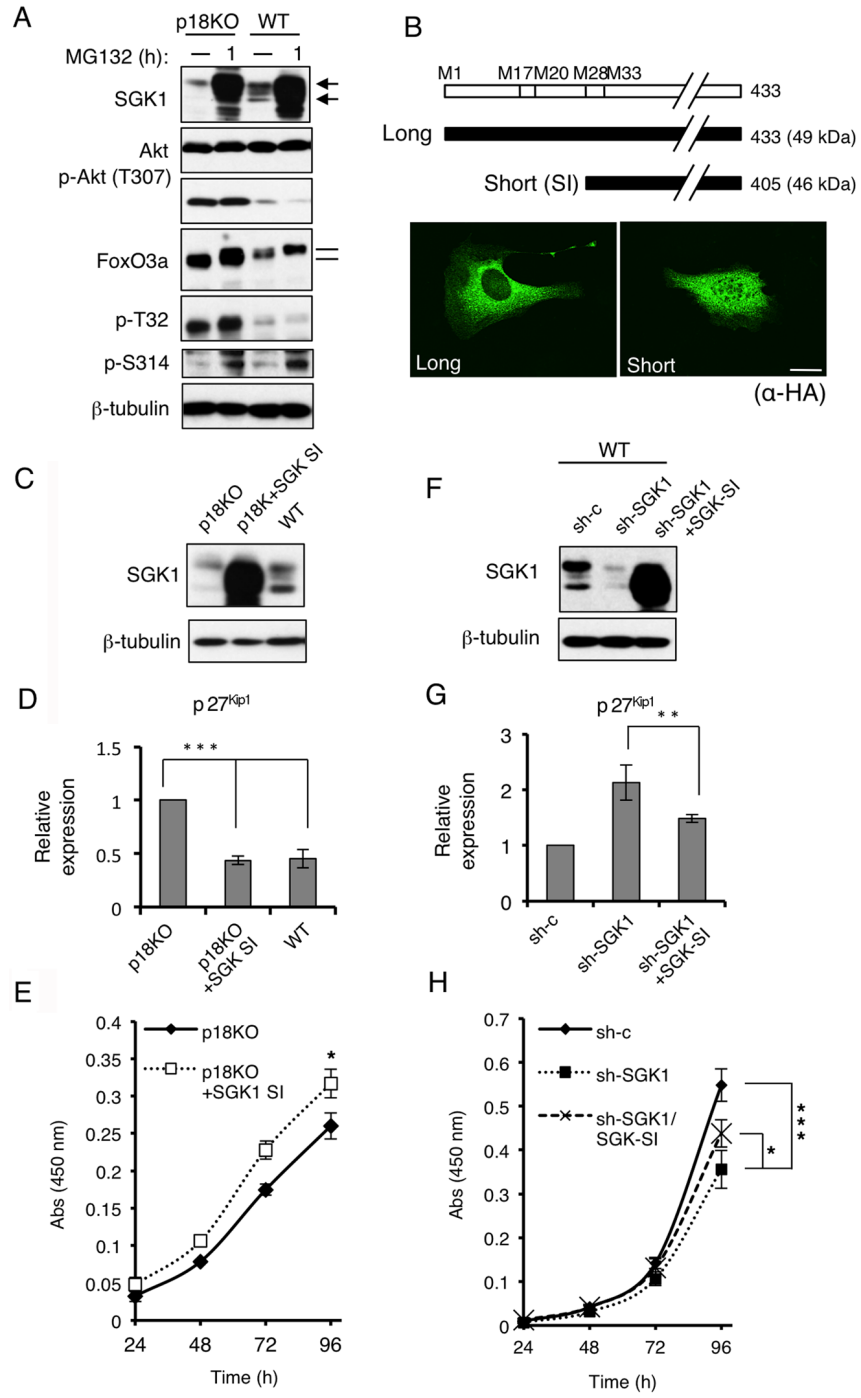
Phosphorylation of FoxO3a Ser314 is mediated by a SGK1 isoform that functions in the nucleus. (**A**) p18KO and WT cells were treated with MG132 for 1 h, and whole-cell lysates were subjected to Western-blot analyses to detect the indicated molecules. Bands corresponding to long and short forms of SGK1 are indicated by arrows. Bars indicate the mobility shift of FoxO3a (**B**) Schematic structures of long and short forms of SGK1 (upper). Immunofluorescence staining to detect HA-tagged long and short forms of SGK1 (lower). Scale bars: 10 µm. (**C**) Western-blot analyses of SGK1 and β-tubulin in p18KO cells, p18KO cells expressing SGK1 SI, and WT cells. Bands corresponding to long and short forms of SGK1 are indicated by arrows. (**D**) Expression of p27^Kip1^ mRNA in cells used in (C) was determined by quantitative real-time PCR. Means±SD were obtained from three independent assays. ***P<0.001 (Student's *t*-test). (**E**) Cell proliferation of p18KO cells and p18KO cells expressing SGK1 SI was analyzed by the WST-1 growth assay over the indicated time course. Means ± SD were obtained from three independent assays. *P<0.05 (Student's *t*-test). (**F**) Western-blot analyses of SGK1 and β-tubulin in WT cells expressing control shRNA (sh-c), SGK1 shRNA (sh-SGK1), or SGK1 shRNA plus sh-resistant SGK1 SI cDNA (sh-SGK1+SGK1 SI) Bands corresponding to long and short forms of SGK1 are indicated by arrows. (**G**) Expression of p27^Kip1^ mRNA in cells used in (F) was determined by quantitative real-time PCR. Means±SD were obtained from three independent assays. **P<0.01 (Student's *t*-test). (**H**) Cell proliferation of cells used in (F) was analyzed by the WST-1 growth assay for the indicated time course. Means ± SD were obtained from three independent assays. ***P<0.001 and *P<0.05 (Student's *t*-test).

Finally, we examined the role of SGK1 in the regulation of the nuclear function of FoxO3a. SGK1 has a very short half-life (<30 min) and is tightly regulated by the ubiquitin-proteasome system [36]-[38]. Indeed, treatment with the proteasome inhibitor MG132 caused dramatic upregulation of SGK1 protein to a similar extent in both p18KO and WT cells (Figure 7A), indicating that synthesis of SGK1 protein is not affected by mTORC1 inactivation. In sharp contrast to the case of SGK1, expression of Akt was not affected by MG132 treatment. Upregulation of SGK1 by MG132 induced a mobility shift of FoxO3a and increased phosphorylation at Ser314, supporting the idea that SGK1 is involved in the phosphorylation of Ser314 in FoxO3a. These results, together with the observation that SGK1 mRNA expression was unaffected (data not shown), suggest that mTORC1 inactivation affects SGK1 protein stability.

As noted above, SGK1 is expressed as isoforms with different N-termini owing to translation initiation at alternative sites [35] (Figure 7B). Western-blot analysis revealed multiple bands corresponding to SGK1; of these, the shortest form was clearly downregulated by mTORC1 inactivation (Figures 5A and 7C). Based on the molecular sizes, the largest and smallest bands correspond to the full-length (isoform e) and the shortest (isoform b) isoforms, respectively (Figure 7B). To discriminate the functions of these isoforms, the corresponding cDNAs were transiently transfected into p18KO cells. The long form was predominantly localized to the cytoplasm, whereas the short isoform was evenly distributed between the cytoplasm and the nucleus (Figure 7B), indicating that the short isoform can function in the nucleus to promote nuclear export of FoxO3a. We then assessed the function of the short form (SGK1 SI) by stably overexpressing it in p18KO cells (Figure 7C). Expression of SGK1 SI significantly suppressed p27^Kip1^ expression to a level comparable with that in WT cells (Figure 7D). Accordingly, cell proliferation was promoted by SGK1 SI expression, although the effect was not large (Figure 7E). On the other hand, shRNA-mediated knockdown of SGK1 promoted p27^Kip1^ expression (Figures 7F and 7G), resulting in significant suppression of cell proliferation (Figure 7H). These effects were rescued by re-expression of an shRNA-resistant SGK1 SI (Figures 7F-7H). These results suggest that the short SGK1 isoform contributes to the regulation of cell proliferation via the FoxO3a-CDKI axis.

## Discussion

**Figure 8:**
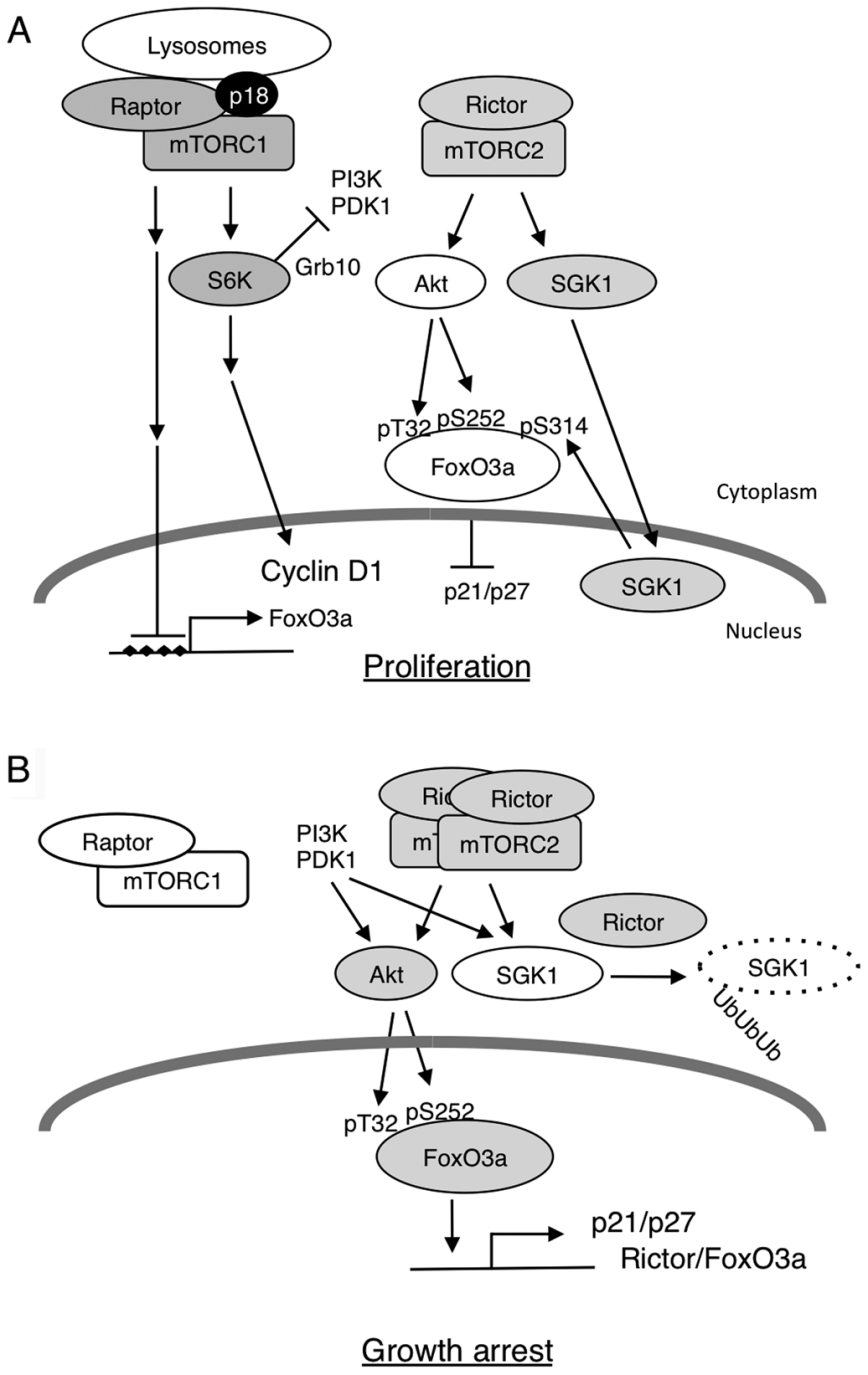
Schematic model of the function of mTOR pathway in the regulation of cell proliferation. (A) When mTORC1 is active. (B) When mTORC1 is inactive.

To dissect the function of mTOR in the regulation of cell proliferation, we investigated the effects of specific inactivation of mTORC1 using p18-deficient cells. Based on our findings, together with those of previous studies, we propose a hypothetical model for mTORC1 function (Figure 8). In WT cells, active mTORC1 promotes protein synthesis to support cell growth, and maintains the activity of Akt at a resting level through negative-feedback regulation via Grb10 [17]. Akt and SGK1 activated by mTORC2 [39] coordinately phosphorylate FoxO3a at Thr32/Ser253 and Ser314, respectively [34]. These modifications result in translocation of FoxO3a from the nucleus, thereby inhibiting expression of CDKIs and ultimately promoting cell proliferation. According to this model, mTORC2 must be constitutively active at an appropriate level to continuously promote cell proliferation. By contrast, when mTORC1 is inactivated, mTORC2 is activated by the induction of Rictor, potentially through the loss of negative-feedback inhibition of the mTORC1 pathway [40]. mTORC2 then induces Akt hyperactivation, resulting in elevated phosphorylation of FoxO3a at Thr32 and Ser253. The induction of Rictor also facilitates ubiquitylation-meditated destruction of SGK1 [36]. Consequently, phosphorylation of FoxO3a at Ser314 is reduced, and FoxO3a retained in the nucleus activates expression of CDKIs, thereby inducing growth arrest. In addition, mTORC1 inactivation alleviates the epigenetic suppression of *Foxo3* gene expression, further enhancing the accumulation of FoxO3a in the nucleus. This model highlights the crucial role of the coordinated action of mTORC1 and mTORC2 in the regulation of cell proliferation via FoxO3a. When mTORC1 is inactivated under physiological conditions, such as starvation, activation of FoxO3a contributes to growth arrest; this process is critical for the survival of cells that are undergoing autophagy to recycle materials and obtain energy. Because we observed that expression and phosphorylation status of FoxO1 were significantly affected by mTORC1 inactivation, it is possible that other FoxO family members also contribute to the growth control via the mTOR pathway.

Previously, we identified p18 as an essential membrane anchor of the p14/MP1 complex, which was isolated as a scaffold for the MAP kinase pathway [21], [30], [31]. However, our analyses in this study using the newly developed p18-deficient fibroblasts, demonstrated that loss of p18 exerts no significant effects on the MAP kinase pathway, suggesting that the role of p14/MP1 is cell context-dependent. In our system, mTORC1 activity is exclusively dependent on the presence of p18 on lysosomes. Therefore, we used this new system to dissect mTORC1-specific functions in the mTOR pathway. The most intriguing finding was that cell proliferation was dramatically suppressed, even though Akt activity was substantially upregulated and FoxO3a was hyperphosphorylated. This apparent discrepancy was resolved by our finding that the reduction in phosphorylation of FoxO3a at Ser314 occurred concomitantly with the downregulation of SGK1 expression.

SGK1 is a member of the SGK family, which consists of SGK1, 2, and 3 [41]. SGK1 is rapidly regulated at the transcriptional level, and by posttranslational modifications, such as phosphorylation and/or ubiquitylation [42]. Like Akt, SGKs are activated by phosphorylation via PDK1 [43] and mTORC2 [39] in response to growth factors; when activated, they phosphorylate various regulatory proteins that control cellular processes such as ion transport in epithelia [42] and cell growth [34]. SGK1 is expressed as multiple isoforms with different N-termini due to translation initiation at alternative sites; these isoforms have different subcellular localizations, functions, and turnover rates [35]. In this study, we found that expression of the shortest form of SGK1 was most clearly downregulated by mTORC1 inactivation. Because the shortest form can be imported into the nucleus, it is likely that this form is preferentially involved in the nuclear export of FoxO3a. We also observed that inhibition of the proteasome dramatically induced accumulation of SGK1, but not Akt, confirming that SGK1 is tightly regulated by the ubiquitin-proteasome system. SGK1 is degraded by several E3 ligases, including Rictor/cullin-1 [36], ERAD systems such as Nedd4-2 [37], and CHIP [38]. Because Rictor is upregulated by mTORC1 inactivation, it is possible that SGK1 is downregulated via the Rictor/cullin-1 E3 ligases when mTORC1 is inactivated, although the potential contribution of other systems cannot be excluded.

We observed that overexpression of a FoxO3a mutant that lacks Ser314 strongly suppressed proliferation of WT cells, demonstrating the crucial role of Ser314 phosphorylation in regulating FoxO3a function in cell proliferation. Overexpression of SGK1 in p18KO cells suppressed the expression of CDKI and promoted cell proliferation, whereas SGK1 knockdown in WT cells induced CDKI expression and suppressed cell proliferation. These results support the idea that SGK1 contributes to the regulation of cell proliferation by phosphorylating FoxO3a. However, these effects of SGK1 expression and knockdown on cell proliferation were relatively moderate compared to the effects of FoxO3a mutants. Therefore, it is possible that although SGK1 takes part in the regulation of FoxO3a, other kinase(s) and/or phosphatase(s), such as PP2A [44], are also required to fully control the phosphorylation status of FoxO3a. Future studies will be directed toward identifying the kinase(s) and/or phosphatase(s) involved. Furthermore, the potential contribution of other types of modifications, such as acetylation [45] and methylation [46], [47], will also need to be examined to elucidate the full regulatory mechanism of FoxO3a.

In this study, we found that expression of FoxO3a is regulated by DNA methylation at a site adjacent to its enhancer region, in a manner that depends on the p18-mTORC1 pathway. To our knowledge, this is the first report to show a functional link between DNA methylation and mTOR signaling. It is possible that mTORC1 signaling regulates the expression of a wider range of genes via epigenetic mechanisms by regulating transcriptional cofactors and enzymes involved in DNA methylation. Because environmental changes, such as starvation, have been implicated in epigenetic changes [48], mTORC1 may also affect DNA methylation status by regulating metabolic pathways that supply donor molecules for DNA methylation reactions. Future studies should address the underlying mechanisms.

Given that SGK1 plays a role in controlling cell proliferation by suppressing FoxO3a function, deregulation of SGK function may be involved in tumor growth. Indeed, several reports have described the contribution of SGK1 and/or SGK3 to cancer progression [41], [49]–[54], and a recent report identified a subset of breast-cancer cell lines that are intrinsically resistant to Akt inhibition due to constitutive upregulation of SGK1 [54], [55]. These observations suggest that SGK might represent a promising therapeutic target in a wide range of cancers in which growth-factor signaling is upregulated, e.g., through mutations in the PI3K pathway.
